# Expanding the use of patient reports about patient-centered care

**DOI:** 10.1186/2045-4015-2-36

**Published:** 2013-09-17

**Authors:** Paul D Cleary

**Affiliations:** 1 Yale School of Public Health, 60 College Street, New Haven, CT 06520, USA

## Abstract

In an informative article on the assessment of patient care experiences, Zimlichman, Rozenblum, and Millenson describe the evolving use of surveys that elicit patient reports about medical care experiences in Israel, a trend that parallels developments in the U.S. This commentary summarizes some of experiences in the U.S. that might inform the development of more consistent and extensive strategies for assessing and promoting patient-centered care in Israel.

More comprehensive patient experience surveys, the results of which would be publicly available, as Zimlichman and colleagues advocate, would facilitate quality improvements, especially if users are provided with support for the use and interpretation of the data. Developing more efficient survey methods will facilitate the broader use of such surveys, although it is important to use methods that yield results that are as representative of the target population as possible and to account for survey mode effects when data are reported. Although the surveys need to be appropriate for the Israeli context, the use of standard questions used in other countries would facilitate comparisons that could help to identify best practices that can be adopted in different settings. Those who work on assessing patient-centered care in the U.S. look forward to learning from the work of their Israeli colleagues.

This is a commentary on http://www.ijhpr.org/content/2/1/35/.

## Commentary

In an informative article on the assessment of patient care experiences, Zimlichman, Rozenblum, and Millenson [1] describe the increasing use of patient assessment of medical care experiences in Israel, a trend that parallels developments in the U.S. In 2001, the U.S. Institute of Medicine published a report stating that a cardinal feature of high quality care is that it should be “patient-centered”, that is, care that is “respectful of and responsive to individual patient preferences, needs, and values and ensuring that patient values guide all clinical decisions” [2]. In the U.S., the Consumer Assessment of Healthcare Providers and Systems (CAHPS®) surveys are now used to assess patient experiences in a wide range of health care settings [3,4]. A major U.S. health plan accrediting organization, the National Committee for Quality Assurance (NCQA), uses CAHPS surveys as one of the measurement tools in its health plan accreditation process and several other national organizations recommend the use of such measures. Also, the recently passed U.S. Patient Protection and Affordable Care Act mandated that the Centers for Medicare & Medicaid Services (CMS) establish a “value-based purchasing” program for hospitals, under which acute care hospitals have their payments for Medicare beneficiaries adjusted, in part based on CAHPS scores.

Although Zimlichman, Rozenblum, and Millenson assert that the Israeli healthcare system is struggling to change in this area, the number of activities related to patient-centered care in Israel is impressive. All four of the non-profit health plans there routinely assess patient experiences and national surveys about care experiences are conducted biannually with representative samples of adults. The assessment of hospital experiences, however, is not done as consistently and there is not yet a coordinated national approach to measuring patient experiences in different settings.

There are substantial system and cultural differences between the U.S. and Israel that are well described by Zimlichman and colleagues. Nevertheless, given the extensive use of patient experience surveys in the U.S., some of the experiences there might be informative for efforts to assess and promote patient-centered care in Israel.

Zimlichman and colleagues state that one of the major differences between health care in the U.S. and Israel is that in Israel health care policy and provision reflect a strong sense of social solidarity, as opposed to the U.S. where there is more competition among providers for patients. Although competition for patients is less likely to motivate improvements in Israel than in the U.S., that might be make it easier in Israel to broadly accept the value of patient assessments.

Financial incentives have increased awareness of, and attention to patient survey data in the U.S., but the increasing focus on financial incentives and competition may have had a negative impact on the way survey data are perceived. For example, because some patients, nurses, and physicians still think of traditional patient satisfaction surveys when patient experience surveys are used, they incorrectly conclude that high scores might be achieved by catering to patient’s desires for unwarranted treatment [5]. CAHPS surveys were designed to assess care quality by asking about important care processes, such as whether information about new medications and post-discharge care were explained clearly, as opposed to relying on general assessments of satisfaction. There is no evidence that providing inappropriate treatment could increase CAHPS scores. In fact, in areas with higher discretionary medical spending, CAHPS scores tend to be lower [6]. One study found that among parents who initially wanted inappropriate antibiotics, those who were told why they did not receive them were more satisfied than those who had simply been given unneeded medication, suggesting that patients want clear communication and participation, not unnecessary care [7]. While overtreatment is often a problem in the U.S., it is unlikely that it is due to a desire to influence patient assessments of their care. Rather, it is likely primarily due to perverse incentives in payment systems. The relative lack of such forces in Israeli health care mean that there is an opportunity for providers to focus on the aspects of the clinical interaction that they value and can improve, rather than worrying about the potential negative consequences of financial incentives.

The ultimate goal of measuring patient experiences is to improve the quality of care. Zimlichman and colleagues note that the results of hospital patient surveys are rarely available publically in Israel. They argue that they should be made available to the public as they are in the U.S. because that might help providers identify areas for improvement and create public pressure for improvement. Certainly, providers should be provided survey results to motivate and guide improvements, even if results are not made public. Making results public, however, could provide further motivation for improvements. Data from the U.S. suggests that public reporting, even in the absence of financial incentives, can lead to improvements [4].

Making survey results available to providers and/or the public is often not sufficient, however, to achieve substantial, sustained improvements. Many factors need to be addressed to facilitate such change. For example, survey data are very different than much of the data used by medical care providers to monitor and improve care. Thus, there often are organizational, professional and data-related barriers to using survey data for quality improvement [8]. Efforts to improve care using patient surveys will be facilitated if providers in Israel are given assistance to address these kinds of issues as they increase the use of patient feedback to improve care processes.

One of the recommendations that Zimlichman and colleagues make is that national patient experience surveys take into account the unique features of Israeli society. Surveys always should be culturally appropriate, but many of the issues assessed in CAHPS surveys, such as communication, are probably salient to virtually all patients and having some comparable measures would facilitate international comparisons [9].

Zimlichman and colleagues specifically recommend that surveys in Israel include assessment of issues such as patient activation and shared decision making. Such issues are important and CAHPS researchers have been actively working on the development of better questions to assess these and related issues. For example, efforts to develop better measures of care processes particularly salient for Patient-Centered Medical Homes resulted in new questions about self-management support and shared decision-making [10]. One of the challenges in developing questions about shared decision making is that it is difficult to develop questions that are specific enough to be interpreted in comparable ways by diverse patients and that are salient for a large sample of diverse patients. Another issue is that although there has been a great deal written about patient activation and shared decision making, these are areas in which care provided at many sites is less than optimal and there is less variation among sites than in other processes of care. This may in part account for why questions about these issues tend to have lower reliability than questions about other issues such as general communication and access [10]. It is important to continue developing and testing questions about these aspects of care, but it is also important to be aware of the difficulties of developing good questions that will be valid and reliable in large samples of patients. Thus, in addition to developing methods that will make it easier and less expensive to survey larger representative samples of patients, it may be useful to assess certain types of issues in more focused samples.

Zimlichman and colleagues state that one of the ways that Israel could learn from experiences in the U.S. is by studying the impact of measuring patient experiences on clinical services. There are many reasons for assessing associations between patient-centered care and other care processes and outcomes. Systems theory suggests that a well-run hospital or clinic could implement procedures and policies that support quality in many areas and processes. However, although different aspects of care quality are sometimes correlated across providers [11-15] and well controlled studies of specific patient groups have shown a positive association between patient-centered care and outcomes [16], findings regarding the associations between patient experiences and other quality measures are mixed [11,12,15,17]. Even different clinical indicators of care quality for the same condition are often not associated across facilities [17].

Although these types of studies are interesting because they help us understand the relationships among different aspects of medical care, patient surveys were never intended to be, and cannot be, a reliable and valid way of assessing the quality of technical care. Furthermore, even if one had a perfect measure of patient-centered care and a perfect measure of technical quality of care, it is quite possible that they would have no, or at least a low association across facilities or providers; they measure different things that are often influenced by different determinants.

Zimlichman and colleagues also suggest that new survey approaches that have the potential of increasing response rates and the timeliness and usefulness of surveys should be explored. This also is an area of great interest in the U.S. and many CAHPS users are interested in using survey methods that are less expensive than mail and telephone surveys, such as Internet surveys, tablets, or interactive voice response [18-20]. The challenge in using these strategies is that for patient survey results to be credible it is important to have data that are as representative of the target population as possible and it often is difficult to obtain reasonable response rates and representative samples with certain types of surveys, such as Internet surveys. Furthermore, it is important to assess the impact of survey mode when data using different methods are used to compare different facilities [21].

One of the exciting opportunities that broader international use of patient surveys provides is comparing the way and extent to which patient-centered care is provided in different countries. Such comparisons may help to identify best practices that can be adopted in different settings [9]. Furthermore, the quality and efficiency of patient surveys will only improve as we accumulate experience in different contexts. Researchers and providers who work on assessing and improving patient-centered care in the U.S. look forward to learning from the work of our Israeli colleagues.

## Competing interests

The author receives grant and contract support for the development and assessment of surveys that assess patient care experiences.

## Authors’ information

PDC is Dean of the Yale School of Public Health.

## Commentary on

Zimlichman, E, Rozenblum, R, Millenson, ML. The road to patient experience of care measurement: lessons from the United States. Isr J of Health Policy Res 2013, 2:35.
